# Chronic, but not sub-chronic, stress increases binge-like alcohol consumption in male and female c57BL6 mice

**DOI:** 10.3389/fnbeh.2022.958342

**Published:** 2022-09-20

**Authors:** William McCarthy, Shama N. Huq, Kristen Allen, Lindsay Scally, Avelina Petri, Madeline Wujek, Benjamin D. Sachs

**Affiliations:** Department of Psychological and Brain Sciences, College of Liberal Arts and Sciences, Villanova University, Villanova, PA, United States

**Keywords:** stress, alcohol, nucleus accumbens, sex differences, mouse

## Abstract

Stress is known to contribute to mental illness and alcohol use disorders, which are highly prevalent and lead to considerable disability. These stress-related disorders are characterized by significant sex differences, which remain poorly understood. Preclinical research comparing the effects of stress in males and females has the potential to provide new insights into the neurobiology of these conditions. The current study compared the effects of chronic and sub-chronic exposure to variable environmental stressors on binge-like alcohol consumption using the drinking-in-the-dark model in male and female c57BL6 mice. The results reveal that chronic, but not sub-chronic, exposure to variable stress increases alcohol intake in both sexes. Stress-induced alterations in gene expression were also compared in the nucleus accumbens, a brain region widely known to play a key role in stress susceptibility and reward processing. Real-time PCR data indicate that chronic, but not sub-chronic, environmental stress leads to downregulation of adenosine 2A (A2A) receptor mRNA. By contrast, sub-chronic stress increased CREB expression, while chronic stress did not. Several sex differences in the effects of stress on gene expression were also noted. Our results demonstrate that reductions in A2A receptor mRNA in the nucleus accumbens are associated with the increased binge drinking of chronically stressed animals, but future work will be required to determine the functional importance of this gene expression change. Continuing to define the molecular alterations associated with stress-induced increases in alcohol intake has the potential to provide insights into the development and progression of stress-related disorders.

## Introduction

Alcohol use disorders (AUDs) are estimated to cost the United States roughly $250 billion annually, approximately three-quarters of which is due to binge drinking (Sacks et al., 2015). In addition to its financial costs, alcohol was a contributing factor in approximately 3% of all deaths in the United States in 2020 (White et al., 2022). Men are almost two times more likely to binge drink than women (Kanny et al., 2018) and have significantly higher rates of AUDs (Schulte et al., 2009). Although stress is thought to increase alcohol consumption in both sexes, women are more likely than men to cite stress coping as a major motivation for drinking (Nolen-Hoeksema and Hilt, 2006; Peltier et al., 2019). The fact that stress promotes alcohol consumption is generally consistent with negative reinforcement models of alcohol intake (Baker et al., 2004) and self-medication and stress reduction theories of excessive alcohol drinking (Powers and Kutash, 1985). Establishing preclinical models of stress-induced escalations of alcohol intake has the potential to provide insights into the mechanisms through which stress can promote or exacerbate AUDs. However, the effects of stress on alcohol consumption in rodents have been reported to be highly variable. This variability is thought to result from several factors, including differences in the type and duration of stress exposure, the time point following stress at which alcohol intake is monitored, sex differences, and the genetic background of animals under investigation (Becker et al., 2011; Mineur et al., 2022). There is also considerable variability in what is considered chronic and sub-chronic stress. For example, 10 days of social defeat stress is often referred to as chronic social defeat (Tsankova et al., 2006; Covington et al., 2011; Lorsch et al., 2021). However, other groups have reported that 15 days of exposure to mild stressors constitutes sub-chronic stress (Ducottet and Belzung, 2004; Ducottet et al., 2004). For the current work, 5 days of stress exposure is being considered sub-chronic, while 21 days of stress exposure is being considered chronic.

Negative reinforcement models and the self-medication hypothesis would both predict that stress paradigms that induce negative affect would also increase alcohol consumption in animals provided voluntary access to alcohol. However, while stress could promote drinking indirectly by inducing negative emotional states, it could also lead to alterations in reward processing, impulsivity, or other processes that could promote drinking independent of negative affect. Indeed, stress has long been known to impact multiple brain regions, including the nucleus accumbens (NAc) (Herman et al., 1982; Krishnan et al., 2007), amygdala (Sapolsky et al., 1984), and frontal cortex (Biggio et al., 1981). Given that the NAc (Sneddon et al., 2021), amygdala (Moller et al., 1997), and frontal cortex (Deckel et al., 1996) are known to regulate drinking behavior and emotional responses (Aggleton, 1993; Barbas, 2000; Lim et al., 2012), stress could potentially impact drinking via multiple mechanisms in several brain regions, but the current study focused on the NAc.

Sex differences in stress susceptibility have been associated with significant differences in gene expression in the NAc (Hodes et al., 2015). Thus, in addition to measuring alcohol intake, the current study examined the expression of several genes that have been implicated in stress susceptibility and/or alcohol intake, including the serotonin 1A (5-HT1A) receptor, cAMP response element-binding protein (CREB), activity-regulated cytoskeleton-associated protein (Arc), neuropeptide Y (NPY), and the adenosine 2A (A2A) receptor. Prior work has shown that decreasing levels of Arc increases alcohol intake in rats, and Arc is thought to be critical for alcohol anxiolytic-like effects (Pandey et al., 2008), which could be particularly important for stress-related alcohol consumption. Similarly, CREB- and NPY-dependent signaling have been hypothesized to play a key role in alcohol intake and anxiety-like behavior (Heilig, 2004; Pandey et al., 2005; Rimondini et al., 2005). The 5-HT1A receptor has long been implicated in anxiety-like behavior and impulse control (Schreiber and De Vry, 1993), and alterations in the levels of 5-HT1A receptors have been detected in individuals who met the criteria for AUDs (Storvik et al., 2009). The A2A receptor has been reported to play a key role in stress susceptibility (Batalha et al., 2013), and it is known to be required for the sedative effects of alcohol (Fang et al., 2017), but whether it plays a role in stress-related alcohol intake is unknown.

While each of the aforementioned genes could potentially regulate alcohol responses independently, these genes encode signaling molecules that interact on multiple levels, and interactions between signaling pathways could also regulate alcohol intake. For example, the 5-HT1A receptor is known to inhibit cAMP production (Weiss et al., 1986) and could therefore negatively regulate CREB activity. By contrast, the A2A receptor is known to stimulate cAMP production (van Calker et al., 1979) and thus could promote CREB activity. The administration of NPY has been reported to increase the activity of CREB (Chance et al., 2000), and NPY is a known target of CREB, with reductions in CREB levels leading to corresponding reductions in NPY (Pandey et al., 2004). In addition to CREB, the activation of the 5-HT1A receptor has also been reported to reduce stress-induced increases in Arc expression (Bader et al., 2014).

The current study was designed to determine whether chronic (21-day) or sub-chronic (5-day) exposure to variable environmental stressors is sufficient to increase binge drinking and lead to the dysregulation of these stress- and alcohol-related genes. The sub-chronic stress paradigm used in the current study (5-day stress, 5DS) was recently shown to increase anxiety-like behavior in both male and female mice (Baugher et al., 2022). Despite leading to largely similar behavioral alterations in male and female mice, 5DS was reported to induce sex-specific changes in gene expression in the NAc (Baugher et al., 2022). The chronic stress paradigm (21DS) used in the current study consists of the same three stressors as 5DS repeated over a 3-week period. Binge-like alcohol intake was measured using the drinking-in-the-dark (DID) paradigm, a well-established test in which mice voluntarily consume intoxicating quantities of alcohol (Thiele et al., 2014).

## Materials and methods

### Animals

This study used 70 male and 70 female c57BL6 mice. A total of two cohorts of 20 mice each (five mice per group, per cohort: male control, female control, male 21DS, and female 21DS) were examined in the chronic stress paradigm, and three cohorts of 20 mice each (five mice per group, per cohort: male control, female control, male 5DS, and female 5DS) were examined in the sub-chronic stress paradigm. Behavioral data from all cohorts were combined, which resulted in five cohorts of control mice being run, but only three cohorts were exposed to 5DS, while two cohorts were exposed to 21DS. Finally, two separate cohorts of 20 mice each were used for the gene expression analyses, one for each of the stress durations. The data from all cohorts were combined for statistical analyses. The mice were approximately 2 months old when stress exposure began. Food and water were available *ad libitum*, except during exposure to the daily stressor and behavioral testing. The mice were housed in groups of three to five per cage in a temperature- and humidity-controlled room on a 12 h light-dark cycle. All studies were performed by protocols approved by the Institutional Animal Care Use Committee (IACUC) at Villanova University.

### Five- and 21-day stress procedures

Variable stress events consisted of three different stressors over either 5 or 21 days. The mice were subjected to restraint stress on day 1, forced swimming on day 2, and tail suspension on day 3. This sequence of stressors was repeated for a total of 5 or 21 days of stress exposure. For restraint stress, the mice were placed inside a ventilated 50 ml conical tube for 1 h inside their home cage. For forced swimming, the mice were placed inside a 4 L Pyrex glass beaker containing 2.5 L of water at 25 ± 1°C for 12 min. For tail suspension, the mice were suspended by the tail with tape for 1 h. Control mice were handled daily and were deprived of food and water during the tube restraint and tail suspension stressors. These stress procedures were adapted from several existing sub-chronic variable stress procedures that use once-daily administration of similar combinations of environmental stressors (Hodes et al., 2015; Eck et al., 2020).

### Drinking in the dark

Drinking-in-the-dark procedures were performed using standard methods (Thiele and Navarro, 2014) with minor modifications. In brief, starting the day after the final stress exposure (either day 6 or 22), the mice were temporarily singly housed starting approximately 2 h into the dark phase to allow for individual alcohol consumption measurements. After 1 h, the mice were given 2 h of access to 20% ethyl alcohol in drinking water. Following the end of a 2 h alcohol access period, the mice were returned to group housing. This procedure was repeated for the first three nights of DID testing. On the fourth and the final night of DID testing, alcohol access was provided for 4 h, instead of 2 h. During the study period, one bottle was found to have leaked in the male control group on night 1, so the data point was excluded from the analyses.

### Gene expression in the nucleus accumbens

For the study, two separate cohorts of mice were killed by cervical dislocation and decapitation 24 h following the final stress exposure (either day 6 or day 22) without having undergone any behavioral testing. Gene expression analyses were performed, as described previously (Sachs et al., 2018). The brains were removed and sectioned into 1 mm-thick slices, and bilateral tissue punches (1.5 mm in diameter) were taken from the NAc. RNA was isolated from the tissue samples using the Ambion PureLink RNA Mini Kit (Thermo Fisher, Waltham, MA, USA) with TRIzol, according to the manufacturer’s instructions. Next, the Thermo Scientific Maxima First Strand cDNA Synthesis Kit was used according to the manufacturer’s instructions to perform reverse transcriptions on the samples. Real-time PCR was performed using a StepOnePlus machine (Applied Biosystems, Waltham, MA, USA) with PowerUp SYBR Green PCR Master Mix (Thermo Fisher). In the male control group, one sample provided gene expression values more than two standard deviations from the mean for three of the five genes examined, and data from that animal was removed as an outlier from all analyses. The primer sequences were obtained from PrimerBank (Wang et al., 2012) and are shown in Table 1. The Ct value for each gene of interest was subtracted from the Ct value for GAPDH for each sample to calculate a delta Ct value. The average delta Ct values for the female control group was calculated, and this delta Ct value was subtracted from each sample delta Ct value to produce a delta delta Ct value. The data were then log-transformed by raising 2 to the power of the negative delta delta Ct value. The transformed values were used for statistical analyses. For comparison, these analyses were repeated using the male control group as the reference value, instead of the female control group, and separately using within-sex normalization. For the within-sex normalization, the average delta Ct value for the male control group was subtracted from all male samples, and the average delta Ct value for the female control group was subtracted from all female samples.

**TABLE 1:** A list of the primer sequences used for real-time PCR.

Primer sequence table		
**Gene name**	**Forward primer**	**Reverse primer**

GAPDH	CAT GTT CCA GTA TGA CTC CAC TC	GGC CTC ACC CCA TTT GAT GT
Arc	GGA GGG AGG TCT TCT ACC GTC	CCC CCA CAC CTA CAG AGA CA
A2A	TTC CAC TCC GGT ACA ATG GC	CGA TGG CGA ATG ACA GCA C
CREB	AGC CGG GTA CTA CCA TTC TAC	GCA GCT TGA ACA ACA ACT TGG
Neuropeptide Y	CTC CGC TCT GCG ACA CTA C	GGA AGG GTC TTC AAG CCT TGT
5HT1A	GCG GTC ACC GAT CTC ATG G	CAG TAC CTG TCT AGC GCG AT

### Statistical analyses

Behavioral data were analyzed by two-way repeated measures ANOVA with sex (male vs. female) and stress (control vs. 5DS vs. 21DS) as between subject factors. The within subject factor was night as animals were tested on four consecutive nights. Mauchly’s test of sphericity was used to test the assumption of sphericity. This assumption was violated, so the Greenhouse–Geisser correction was used for within-subjects repeated measures analyses. In addition, separate two-way ANOVAs were performed for each night of drinking behavior. For these analyses, Tukey’s *post hoc* tests were performed to identify individual group differences in the between-subjects factors. Gene expression data and alcohol intake on each of the four nights were also analyzed by two-way ANOVA with sex and stress as factors, followed by Tukey’s or Bonferroni’s *post hoc* tests. *P*-values less than 0.05 were considered significant, and JMP software, version 16, (SAS, Cary, NC, USA) and SPSS software, version 26 (IBM, Armonk, NY, USA), were used for statistical analyses.

## Results

### Drinking in the dark

**FIGURE 1:**
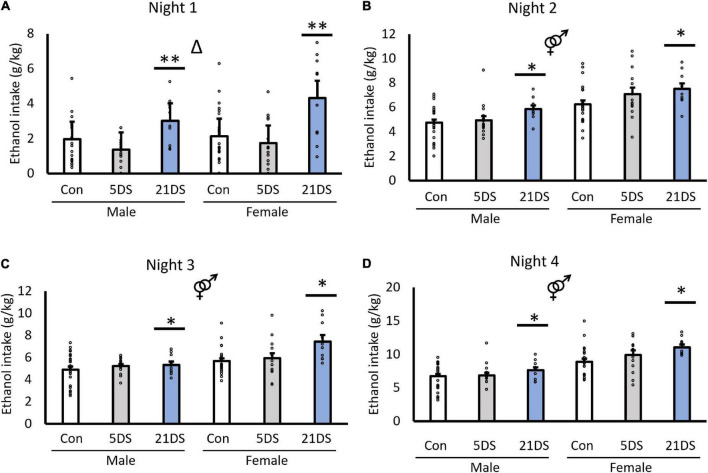
Chronic stress (21DS), but not sub-chronic stress (5DS), significantly increases binge-like alcohol consumption in male and female mice. Nightly alcohol intake using the drinking-in-the-dark test is shown for night 1 **(A)**, night 2 **(B)**, night 3 **(C)**, and night 4 **(D)**. *N* = 25–26 for the male control group, 24 for the female control group, 16 for the male 5DS group, 14 for the female 5DS, 10 for the male 21DS group, and 10 for the female 21DS group. Δ indicates a main effect of stress, and the male/female sign indicates a main effect of sex by two-way ANOVA (*p* < 0.05). *Significantly different from the control group, and ^**^significantly different from both the control and the 5DS groups by Bonferroni’s *post hoc* test.

In the DID, between-subjects analysis revealed significant effects of stress [*F*_(2,94)_ = 9.819, *p* < 0.001, Figure 1] and sex [*F*_(1,94)_ = 42.157, *p* < 0.001, Figure 1]. For the sex effect, female mice consumed significantly more alcohol per body weight than male mice overall. For the stress effect, *post hoc* analyses revealed that the 21DS group consumed significantly more alcohol than the 5DS and control groups (*P* < 0.002 by Bonferroni’s *post hoc* test), but there were no significant differences between the 5DS and control groups. There was no significant sex–stress interaction in the between-subjects analysis [*F*_(2,94)_ = 1.639, *p* = 0.200, Figure 1]. Repeated measures within-subject analysis revealed that alcohol consumption did increase across days in the DID test [*F*_(1.7, 159.4)_ = 303.236, *p* < 0.001], although this effect may be driven by the increased alcohol access time on night 4. A significant two-way interaction between day and sex was also observed, in which female mice increased their drinking more than male mice over the course of the experiment (*F*_(1.7,159.4)_ = 12.717, *p* < 0.001). There was also a trend toward a stress–day interaction [*F*_(1.7,159.4)_ = 2.311, *p* = 0.071], but this did not quite reach statistical significance. Separate two-way ANOVAs for each of the four nights of drinking revealed significant effects of stress on each of the four nights and significant effects of sex on nights 2–4 (Figures 1B–D), but not on night 1 (Figure 1A). Tukey’s *post hoc* tests revealed that the female 21DS group drank significantly more on night 1 than any other group, except the male 21DS group, but there were no significant differences between any of the other groups. On night 2, Tukey’s *post hoc* tests revealed no significant differences between the stress conditions within either sex. On night 3, the female 21DS group consumed significantly more alcohol than any of the three male groups and the female control group, but there were no significant differences between the female 5DS group and the female 21DS group. Finally, on night 4, females in both the 5DS and 21DS groups consumed significantly more alcohol than the female control group, but no significant differences between stress conditions were observed in male mice.

When male and female mice were analyzed separately, stress significantly increased alcohol intake in female mice on nights 1 [*F*_(2,47)_ = 7.71, *p* = 0.001], 3 [*F*_(2,47)_ = 5.316, *p* = 0.008], and 4 [*F*_(2,47)_ = 3.78, *p* = 0.03], but not on night 2 [*F*_(2,47)_ = 2.554, *p* = 0.089]. In male mice, the effects of stress did not reach statistical significance on any of the nights, although trends were observed for nights 1 [*F*_(2,51)_ = 2.96, *p* = 0.061] and 2 [*F*_(2,51)_ = 2.947, *p* = 0.062].

### Gene expression

**FIGURE 2:**
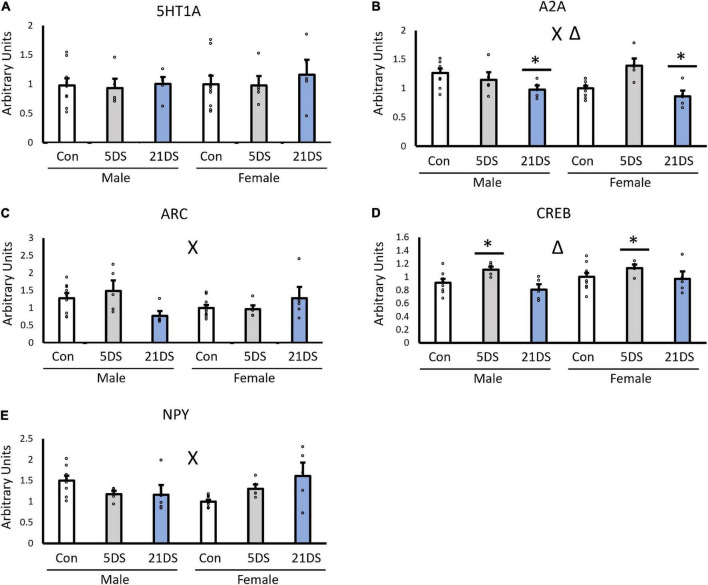
Impact of chronic stress (21DS) and sub-chronic stress (5DS) on the expression of the 5-HT1A receptor **(A)**, the A2A receptor **(B)**, Arc **(C)**, CREB **(D)**, and NPY **(E)** in the NAc of male and female mice. *N* = 9 in the male control group, *N* = 10 in the female control group, and *N* = 5 in all other groups. Δ Indicates a main effect of stress and *X* indicates a sex–stress interaction by two-way ANOVA (*p* < 0.05). *Significantly different from the other two stress conditions by Tukey’s *post hoc* test.

When the female unstressed group was used as the reference/control group, no significant effects of sex were observed for the expression of 5-HT1A (Figure 2A, *p* = 0.563), A2A (Figure 2B, *p* = 0.508), Arc (Figure 2C, *p* = 0.472), CREB (Figure 2D, *p* = 0.111), or NPY (Figure 2E, *p* = 0.825). However, stress significantly impacted the expression of A2A [*F*_(2,38)_ = 7.601, *p* = 0.002, Figure 2B] and CREB [*F*_(2,38)_ = 5.458, *p* = 0.009, Figure 2D]. For the A2A receptor, the 21DS group exhibited lower levels of A2A expression than the control group (*p* = 0.04) and the 5DS group (*p* = 0.002). No significant differences were observed between the 5DS and the control group for A2A expression (*p* = 0.245). For CREB, the 5DS group had higher levels of CREB mRNA than either the control group (*p* = 0.047) or the 21DS group (*p* = 0.01). In addition, significant sex–stress interactions were observed for A2A [*F*_(2,38)_ = 5.227, *p* = 0.011, Figure 2B], Arc [*F*_(2,38)_ = 4.334, *p* = 0.021, Figure 2C] and NPY [*F*_(2,38)_ = 7.133, *p* = 0.009, Figure 2D]. Regarding the sex–stress interaction for A2A, Tukey’s *post hoc* analysis revealed that the female 5DS group exhibited increased A2A compared to female controls (*p* = 0.0147) and the female 21DS group (*p* = 0.0029). For the sex–stress interaction for Arc, 21DS tended to decrease Arc expression in male mice, but it tended to increase Arc expression in females, but no significant group differences were observed by Tukey’s *post hoc* test. For the sex–stress interaction for NPY, both 5DS and 21DS tended to increase NPY expression in female mice but to decrease it in male mice. Tukey’s *post hoc* analyses revealed that both the female 21DS group (*p* = 0.026) and the male control group (*p* = 0.032) exhibited higher levels of NPY than the female control group.

When the unstressed male group was used as the control group, the observed results were largely similar, but there were several notable differences. For A2A, the sex–stress interaction was again statistically significant [*F*_(2,38)_ = 4.99, *p* = 0.013], but unlike female controls, no significant effect of stress was observed. For Arc, the sex–stress interaction was again significant when using the unstressed male mice as the control group, instead of female mice [*F*_(2,38)_ = 4.12, *p* = 0.0252]. However, a significant effect of stress was also observed [*F*_(2,38)_ = 4.85, *p* = 0.014], an effect that was not statistically significant when unstressed female mice were used as the control group. For 5-HT1A, NPY, and CREB, all significant findings with female controls were also found to be significant with male controls, and no additional main effects or interactions were observed with the change in the normalization method.

## Discussion

The current results indicate that chronic, but not sub-chronic, exposure to variable stressors is sufficient to increase binge-like alcohol consumption in both male and female mice. The fact that the same sub-chronic stress paradigm (5DS) that fails to promote binge drinking does significantly increase anxiety-like behavior and forced swimming immobility (Baugher et al., 2022) suggests that stress-induced negative affect is not sufficient to increase binge-like alcohol consumption. Importantly, anxiety-like phenotypes following 5DS have only been reported on days 7 and 8 (the days preceding nights 2 and 3 in the current study), whereas alterations in forced swimming behavior were reported on days 8 and 9 (prior to nights 3 and 4 in the current study). While we are confident that the forced swimming alterations induced by 5DS would be observed at earlier time points, it is less clear how long the increased anxiety of 5DS-exposed mice persists following 5DS exposure. If 5DS-induced anxiety-like phenotypes are transient, this could contribute to the lack of significant increase in alcohol consumption following 5DS.

In addition, it is unclear whether the 4-day DID procedure used here provides mice with enough time and experience with alcohol to learn that alcohol consumption can alleviate anxiety-related phenotypes. If not, increased binge-like drinking to reduce negative affect would not be expected. Regardless, the fact that 21DS-exposed animals tested for 4 days in the DID paradigm do display increased alcohol intake suggests that an extensive history of alcohol intake is not an absolute requirement for stress-induced increases in binge-like drinking. Nonetheless, it remains to be determined whether 5DS would increase DID alcohol consumption in animals with a longer history of alcohol intake. The reasons why 21DS significantly increased drinking, while 5DS did not, have not been conclusively established. Prior work with the 5DS model has shown that it is sufficient to increase corticosterone levels in mice (Baugher et al., 2022), and that while 21DS may be more effective than 5DS at increasing corticosterone levels to promote drinking, this has not been tested. Alternatively, chronic stress, but not sub-chronic stress, could be sufficient to promote dysregulation of neural circuits that control alcohol intake [for a review of relevant circuits, see Lovinger and Alvarez (2017)]. Indeed, some of our gene expression data (discussed later) are consistent with this possibility. Future research comparing the cellular and molecular alterations induced by 21DS vs. 5DS across multiple brain regions has the potential to provide new insights into some of these potential pathological processes.

The current findings add to a large body of research examining the effects of stress on alcohol intake in rodent models, in which many conflicting findings have been reported [reviewed in Becker et al. (2011)]. In general, most mouse studies report that acute environmental stress either has no effect or reduces alcohol intake on days in which environmental stress is administered, but stressor-specific changes have been observed at later time points. For example, short-term exposure to several of the stressors employed here, including restraint (30 min) and tail suspension (6 min), has been reported to reduce alcohol intake in a prior study. Specifically, acute restraint induced a reduction in alcohol intake that lasted for 2 days following stress exposure, while tail suspension suppressed alcohol intake only on the stress exposure day (Cozzoli et al., 2014). Interestingly, another prior study has reported that female CD1 mice that are prone to excessive immobility in the tail suspension test drink more alcohol than female mice that exhibit less immobility in this test, but no such relationship was observed in male mice (Pelloux et al., 2005), which suggests that the factors leading to increased alcohol intake differ in male and female mice. Unlike restraint and tail suspension, a single exposure to 30 min of predator odor stress increased alcohol consumption, starting the day after stress exposure in female mice and starting 2 days after stress exposure in male mice (Cozzoli et al., 2014). The reasons why acute exposure to different environmental stressors leads to different effects on alcohol intake remain to be established.

Similar to acute stressors, the effects of chronic environmental stressors on alcohol consumption in adult rodents also appear to depend on the specific stress paradigm employed and the sex and genetic background of animals being studied. For example, unpredictable chronic stress (11 days) has been reported to increase binge-like alcohol intake in male mice, but not in female mice (Quadir et al., 2019). Chronic mild stress (7 weeks) has been reported to have variable effects on DID alcohol consumption in female mice taken from crosses between mice on a c57BL6/J or DBA background, with stress reducing binge-like alcohol intake in most strains but increasing it in others (Mulligan et al., 2019). Chronic repeated predatory stress has been reported to increase the consumption of aversive alcohol solutions containing quinine in male c57BL6/J mice, but not in female mice (Shaw et al., 2020).

Although there is considerable variability in the effects of environmental stress on alcohol intake overall, social stressors have been more consistently reported to increase alcohol intake. For example, early-life maternal deprivation has been reported to increase alcohol consumption in adult male and female rats, and this effect has been reported to be enhanced by restraint stress during adulthood (Penasco et al., 2015). In addition, social defeat stress is sufficient to increase alcohol consumption in male mice (Kudryavtseva et al., 2006; Norman et al., 2014; Rodriguez-Arias et al., 2014; Newman et al., 2018; Montagud-Romero et al., 2021; Reguilon et al., 2021a,b) and female mice (Newman et al., 2021).

To our knowledge, the current study is the first to examine potential sex differences following 5 or 21 days of exposure to variable environmental stressors. No significant sex–stress interaction was observed, and thus, we can only conclude that stress increased alcohol intake independent of sex. While the *post hoc* tests reveal a significant effect of stress in female mice, but not in male mice (without a sex–stress interaction), this does not provide strong support to arrive at a conclusion that stress differentially impacts the sexes (Nieuwenhuis et al., 2011). Nonetheless, given the apparent trend toward greater effects in female mice, it is possible that future studies with larger sample sizes or intermediate stress durations could still reveal significant sex differences in susceptibility to stress-induced binge drinking.

Although exposure to 5DS during adulthood had no effect on binge-like alcohol intake, future research should evaluate whether exposure to 5DS during adolescence would increase alcohol intake during adolescence or in adulthood. Several prior studies have reported that chronic stress during adolescence can promote increased alcohol intake in adulthood. For example, 25 days of chronic variable social stress starting on post-natal day 25 has been shown to increase DID alcohol intake 1 week later (Caruso et al., 2018). Chronic variable stress in adolescence has also been reported to increase alcohol consumption in adult male and female mice (Lopez et al., 2011). However, whether sub-chronic stressors during adolescence have similar effects in male or female mice remains unknown.

Our gene expression data reveal several distinct molecular alterations in the NAc following 5DS vs. 21DS. In particular, the reduction in A2A expression in 21DS-exposed mice compared with 5DS and control animals could have important implications for binge-like alcohol intake. Indeed, given that the hypnotic effects of alcohol have been argued to be mediated by the A2A receptor (Fang et al., 2017), it is possible that reductions in the expression of this receptor would render mice less sensitive to the sedative-like effects of alcohol, which could promote increased intake (Schuckit, 1994). Indeed, in the absence of stress, A2A receptor agonists have been reported to reduce alcohol intake, while antagonists were reported to increase alcohol consumption (Thorsell et al., 2007; Micioni Di Bonaventura et al., 2012). Consistent with this, mice that completely lack the A2A receptor have been reported to exhibit increased alcohol consumption (Naassila et al., 2002). However, there may be multiple additional mechanisms through which 21DS increases alcohol intake. Future research would be required to determine whether any of the molecular differences observed here are causally related to the increased binge alcohol consumption that we observe in the mice exposed to 21DS, but not in the mice exposed to 5DS. Nonetheless, our results demonstrate that chronic and sub-chronic stressors exert differential effects on gene expression, and these different molecular adaptations could underlie the distinct behavioral responses to chronic and sub-chronic stress. Interestingly, several sex–stress interactions on gene expression were observed (for A2A, Arc, and NPY). Given that male and female mice exhibited largely similar behavioral responses to 21DS and 5DS, this may reflect an example of sexual convergence, in which similar behavioral phenotypes arise as the result of distinct neurobiological processes in male and female mice. Indeed, we had previously noted several additional sex differences in 5DS-induced gene expression changes in the NAc, despite the fact that 5DS was shown to induce largely similar effects on depression- and anxiety-like behavior in both sexes (Baugher et al., 2022).

Regarding the gene expression analyses, slight differences in results of statistical tests depending on whether male or female mice were designated as the “control” group suggest that control group selection can significantly impact data interpretation and the conclusions of these types of studies. Importantly, the three significant stress–sex interactions in gene expression that were identified with female controls (Arc, NPY, and A2A) were also statistically significant with male controls. However, the fact that the significant effects of stress observed for Arc and A2A were dependent on the sex used to normalize suggests that these effects may not be particularly robust. By contrast, the main effect of stress on CREB expression was observed regardless of which sex was set as the control group. Instead of using a single sex as a control group and comparing across sexes, it could also be argued that each sex should be normalized to the control group of the corresponding sex, although this prevents meaningful comparisons across sex. Despite this limitation, within-sex normalization can provide insights into whether there are differences in the magnitude of stress-induced changes between the sexes. When we employed within-sex normalization, all three statistically significant stress–sex interactions identified using a single sex as the control group remained significant. However, within-sex normalization also identified a significant sex–stress interaction for the expression of CREB [*F*_(2,38)_ = 4.44, *p* = 0.02], which was not determined to be significant using either of the other two normalization methods.

Overall, the current data provide new insights into the effects of chronic and sub-chronic stressors on alcohol consumption. The observed sex differences in molecular alterations induced by stress in NAc could have important implications for sex-specific behavioral responses to stress, although the only behavior examined in the current study was impacted by stress similarly in both sexes. Continuing to define the behavioral and molecular alterations induced by stress in male and female mice has the potential to reveal sex-specific pathological processes that could eventually be targeted to reduce the burden of stress-related disorders.

## Data availability statement

The raw data supporting the conclusions of this article will be made available by the authors, without undue reservation.

## Ethics statement

This animal study was reviewed and approved by Villanova University Institutional Animal Care and Use Committee.

## Author contributions

WM, SH, and BS contributed to the conceptualization and design of the study and performed the statistical analyses. WM, SH, KA, LS, AP, and MW performed the experiments, collected the data, and contributed to revising the manuscript. BS wrote the initial draft. All authors contributed to the article and approved the submitted version.
